# Time Matters: Possible Predictors of the Number of Appointments During the First Year After Hearing Aid Fitting

**DOI:** 10.1044/2026_AJA-25-00240

**Published:** 2026-04-15

**Authors:** Shannon C. McCabe, Kaitlyn Ramirez, Sara Dyer, David Velenovsky, Tom Muller, Megan J. Kobel

**Affiliations:** a Department of Speech, Language & Hearing Sciences, The University of Arizona, Tucson

## Abstract

**Purpose:**

Follow-up care after hearing aid fitting is recommended, yet clinicians lack data on the expected number of follow-up appointments needed and which patients require more support. This study aimed to characterize follow-up service utilization during the first year after hearing aid fitting and to determine whether patient, device, or cost-related factors may predict the number of follow-up appointments.

**Method:**

A retrospective chart review was conducted on 223 adult patients who received hearing aids at a university-affiliated audiology clinic between May 2022 and April 2023. Demographic, audiological, device-specific, and cost-related data were extracted. The number of follow-up appointments over 1 year was used as the primary outcome. Negative binomial regression analyses were performed to identify predictors of follow-up service utilization.

**Results:**

Patients attended an average of 4.1 follow-up appointments (range: 1–16), with most attending three to five visits. No significant effects were observed for gender, degree of hearing loss, or language. Effects of age and prior hearing aid experience were identified with older patients and those with over 10 years of prior hearing aid use attending more appointments. Device factors, including dispensing of accessories and dispensing of standard-level technology, were associated with increased follow-up utilization. Patients with higher out-of-pocket (OOP) costs or no insurance coverage attended more appointments, though cost and technology level exhibited interdependencies.

**Conclusions:**

Overall, most adult hearing aid patients attended approximately three to five appointments over the first year, and most demographic and audiometric factors did not predict follow-up appointment attendance. However, on average, patients with higher OOP costs and those with an accessory attended more appointments. Clinicians can use these data to provide evidence-based counseling to patients and to inform clinical decision making.

Age-related hearing loss is common and can negatively affect communication, social participation (Prieur Chaintré et al., 2024), and quality of life (Borre et al., 2023). Hearing aids are effective at improving communication and health-related quality of life (Ferguson et al., 2017), but only a minority of those with hearing loss obtain hearing aids and use them consistently (Powers & Rogin, 2019). Access to appropriate follow-up clinical support can promote consistent use of amplification after fitting (Barbosa et al., 2015; Solheim et al., 2012) and can improve short-term satisfaction (Solheim et al., 2018). Hearing care professionals can emphasize the importance of follow-up care facilitating adherence to follow-up recommendations (Khan et al., 2023; Solheim et al., 2012). The number and duration of follow-up appointments can vary substantially across patients, making it difficult for clinicians to provide evidence-based counseling about expected follow-up care and for clinics to plan staffing and caseloads. Without quantitative information on follow-up patterns, clinicians must rely on anecdotal experience when counseling patients and allocating time for post-fitting care.

Most prior work on hearing aid management has focused on barriers that may impact adherence to follow-up, such as financial constraints, transportation, and work commitments (Iwahashi et al., 2015; Solheim et al., 2012, 2018). Past research suggests an interaction between device technology and satisfaction (Hausladen et al., 2022; Newman et al., 1993) and an influence of device cost on pursuing amplification (Newman et al., 1993; Ramachandran et al., 2011). However, little is known about the frequency of patient follow-up visits in routine clinical practice, or which patient, device, or cost-related factors are associated with greater follow-up needs.

The primary purpose of this study was to determine the variability in the number of appointments devoted to follow-up during the first year after hearing aid fitting and to identify if there are any patient, device, or cost factors that may predict the need for additional appointments. These data can help professionals appropriately counsel patients about expected follow-up care and may also inform decisions about scheduling, resource allocation, and service delivery models in adult hearing aid practices.

## Method

A retrospective chart-review of hearing aid patients seen in The University of Arizona Speech, Language, and Hearing (SLHS) Clinic was completed. Approval was obtained through The University of Arizona Institutional Review Board (IRB #00004475; 6/3/2024), and all research activities followed IRB and Health Insurance Portability and Accountability Act compliance. As this was a retrospective chart review, a waiver was granted for written informed consent owing to the minimal-risk nature of the study and use of de-identified data.

Adult patients were reviewed and included if they had hearing aids dispensed in the SLHS Clinic from May 01, 2022, to April 30, 2023. Charts were screened for eligibility and patient, demographic, audiologic, device-specific, and payment-related variables were extracted and coded as described in the Variables Extracted section for each eligible patient. All appointments occurring within 12 months of the fitting date were then reviewed, and the total number of post-fitting appointments was summed to create the primary outcome.

The initial search yielded 283 patients for review. After initial screening, 30 were excluded from further analyses as either (a) the patient was under 18 years of age, (b) hearing aids were not obtained in the SLHS clinic, or (c) dispensing appointment did not represent the patient obtaining a new set of hearing aids (e.g., replacing a lost hearing aid). This yielded 253 patients, which were reviewed for further detail.

### Variables Extracted

From the identified charts, information was extracted to characterize patient demographics, device information, and payment-related factors. For each participant, age in years, gender, and preferred language were recorded. Poorer ear pure-tone average (PTA; using air conduction 1000, 2000, and 4000 Hz) was calculated from the most recent audiogram utilized for the hearing aid fitting. No response at the limits of the audiometer was coded as 120 dB HL. Scores from word recognition (WRS) in quiet were recorded. Past hearing aid experience was categorized as (a) no previous experience, (b) 1–5 years, (c) 5–10 years, and (d) 10 or more years.

Device-specific information for each participant was also extracted. For each fitting, level of technology (basic, standard, advanced, premium) and type of battery (disposable, rechargeable) were recorded. Any accessories dispensed (e.g., remote microphone, TV connector) were categorically noted as binary yes or no. Style of device was characterized as being receiver-in-the-canal (RIC), behind the ear, or in-the-ear (ITE). As there were only nine completely-in-the-canal devices, these were coded as ITE.

To assess the influence of patients' out-of-pocket (OOP) cost and insurance coverage, total cost for devices was recorded from accounting invoices. Cost of earmolds and accessories were excluded from total OOP cost. Insurance coverage and enrollment in specific programs to supplement cost (i.e., vocational rehabilitation, Southern Arizona Hearing Aid Bank) were recorded.

To quantify professional service utilization, all appointments for 1 year after the fitting appointment were recorded. Appointments included (a) fitting, (b) follow-up (appointments for adjustment and counseling), (c) troubleshooting (appointments due to hearing aid malfunction), and (d) drop-off for in-house or manufacturer repair. The initial selection appointment was not counted, as all patients had to complete this appointment at the clinic to be included in the analyses. Due to the small sample size, type of appointment was not differentiated in the analyses.

In the SLHS clinic, fitting appointments are typically scheduled for 90 min while follow-up and troubleshooting appointments are scheduled for 30 min. Real-ear verification was used for all fittings. Post-fitting validation measures were inconsistently collected; thus, they were not included in the final data set. The number of follow-up appointments was chosen as our primary metric of follow-up services. Currently, the SLHS provides services using a bundled pricing structure in which one cost covers both devices and professional services, except for self-pay patients (i.e., no insurance coverage). These patients paid in a semi-unbundled manner, with a cost for the hearing aid separate from the cost for a 3-year service agreement. Total cost for both hearing aids and service agreement was included in total OOP cost for all patients.

### Analyses

To assess the impact of patient, device, and payment factors on follow-up service utilization, negative binomial regression analyses were completed as exploratory analyses suggested overdispersion (i.e., variance greater than mean) of our dependent variable. For all analyses, the total number of appointments was included as the dependent variable. To assess patient factors, age in years, gender, prior amplification experience, PTA of the poorer hearing ear, WRS from the poorer hearing ear, and preferred language were included as independent variables (see Table 1). Analyses assessing device-specific impact on service utilization, level of technology, style of device, and binary use of any accessory were included (see Table 2).

**Table 1.** Patient demographic and audiologic characteristics and regression-based associations with total number of follow-up appointments.

Demographics	*M* (*SD*)	*n* (%)	β [95% CI]	*z*	*p* value
Age	76.08 (11.87)		**.007 [.001, .013]**	**2.13**	**.0033**
Gender					
Female		113 (50.7%)	Reference		
Male		110 (49.3%)	.069 [−.710, .208]	0.98	.329
Experience					
None		109 (48.9%)	Reference		
1–5 years		35 (15.7%)	.196 [−.001, .391]	1.82	.069
6–10 years		30 (13.5%)	.115 [−.103, .332]	1.03	.301
10+ years		49 (22.0%)	**.263 [.702, .001]**	**2.25**	**.007**
Worse ear PTA	62.77 (21.14)		−.003 [−.007, .001]	−1.66	.097
Worse ear WRS	54.76 (33.18)		.0006 [−.002, .002]	0.56	.574
Language					
English		193 (86.5%)	Reference		
Spanish		29 (13.0%)	−.120 [−.337, .096]	−1.09	.276
Other		1 (0.4%)	−.768 [−2.194, .659]	−1.05	.292

*Note.* Values are *n* (%) for categorical variables and *M* (*SD*) for continuous variables. Bolded values indicate statistical significance (*p* < .05) in regression analyses. CI = confidence interval; PTA = pure-tone average; WRS = word recognition score.

**Table 2.** Device characteristics for patients included in the analysis and regression-based associations with total number of follow-up appointments.

Device information	*n* (%)	β [95% CI]	*z*	*p* value
Style				
RIC	131 (58.7%)	Reference		
BTE	70 (31.4%)	.001 [−.155, .158]	0.02	.986
ITE	22 (9.9%)	.088 [−.149, .326]	0.73	.465
Technology level				
Basic	27 (12.1%)	Reference		
Standard	85 (38.1%)	**.294 [.053, .536]**	**2.39**	**.017**
Advanced	72 (32.3%)	.136 [−.110, .383]	1.08	.279
Premium	39 (17.5%)	.043 [−.239, .325]	0.30	.765
Battery				
Disposable	36 (16.2%)	Reference		
Rechargeable	186 (83.8%)	.069 [−.125, .264]	0.7	.485
Accessories				
None	183 (82.1%)	Reference		
1 or more	40 (17.9%)	**.216 [.035, .397]**	**2.34**	**.019**

*Note.* Values are *n* (%) for categorical variables and *M* (*SD*) for continuous variables. Bolded values indicate statistical significance (*p* < .05) in regression analyses. CI = confidence interval; RIC = Receiver-in-the-canal; BTE = Behind-the-ear; ITE = In-the-ear.

Payment-related factors were quantified in two separate analyses. First, the impact of insurance coverage was assessed through classification of payments as having (a) full coverage, indicating that there were no OOP costs; (b) partial coverage for patients with insurance benefits, which did not cover the full cost of devices; and (c) no coverage and the full cost of devices was OOP (see Table 3).

**Table 3.** Insurance coverage and out-of-pocket (OOP) costs and associations with total number of follow-up appointments.

Payment	*n* (%)	Mean (min–max)	β [95% CI]	*z*	*p* value
Coverage					
Full coverage	84 (37.7%)	$0	Reference		
Partial coverage	102 (45.7%)	$1,546 ($199–$4,998)	.151 [−.004, .306]	1.91	.056
No coverage	37 (16.6%)	$3,368 ($1,380–$5,936)	**.243 [.044, 442]**	**2.39**	**.017**
Total OOP cost					
$0	84 (37.7%)	$0 ($0–$0)	Reference		
$1–$1,000	33 (14.8%)	$685 ($199–$1,380)	−.068 [−.293, .156]	−0.6	.551
$1,001–$2,000	54 (24.2%)	$1,561 ($1,198–$2,000)	**.213 [.037, .389]**	**2.37**	**.018**
$2,000+	52 (23.3%)	$3,396 ($2,023–$5,936)	**.271 [.095, .446]**	**3.02**	**.002**

*Note.* Values are *n* (%). Bolded values indicate statistical significance (*p* < .05) in regression analyses. min = minimum; max = maximum; CI = confidence interval.

As there was substantial overlap in OOP costs between those with partial and no coverage (discussed below in the Results section), subsequent analyses investigated the OOP cost on service utilization through arbitrarily categorizing costs as (a) no OOP costs (i.e., $0), (b) $1–$1,000, (c) $1,001–$2,000, and (d) $2,000+ (see Table 3). Postestimation pairwise comparisons were conducted for categorical predictors that were statistically significant in negative binomial regression models, with *p* values adjusted using a Holm–Bonferroni correction. To assess differences in distribution between two variables, chi-squared tests of independence were completed. For all analyses, a *p* value of .05 was considered significant and *p* values represent corrected *p* values as appropriate. All analyses were completed using Stata (Version 18.5).

## Results

A total of 253 records were reviewed. Nine patients received coverage through vocational rehab and were excluded from further analyses as these patients were required to schedule a minimum number of follow-up appointments. Patients who received devices through the Southern Arizona Hearing Aid Bank were also excluded from analyses (*n* = 19) as these patients had a different pricing structure than the other participants. This yielded 223 total patients included in analyses. Patients attended on average 4.11 (*SD* = 2.23) follow-up appointments ranging from one to 16 appointments, with only seven attending 10 or more appointments (see Table 1).

### Patient Factors

Demographic information is outlined in Table 1. Included patients were on average 76 years of age (range: 20–102; *Mdn* = 77 years). Approximately half of the patients (48.6%) were new amplification users, whereas 22% were long-time users with 10 or more years of previous experience. Overall, 86.5% of patients were English speaking, 13% were Spanish speaking, and only 0.4% of patients spoke another language. On average, patients had a PTA of 6.8 dB HL (range: 26.5–120) and 60% WRS (range: 0%–100%) in the worse hearing ear.

Regression analyses did not identify a significant impact of gender, PTA, WRS, or language on number of appointments scheduled (*p* > .05; see Table 1). A significant, yet small, effect of age (β = .007, *p* = .003) was found indicating that, in general, older patients attended more follow-up appointments. Also, an impact of past experience was identified for patients with 10 or more years of past hearing aid experience (β = .263, *p* = .007). Patients with 10 or more years of experience attended, on average, one more appointment than new users (4.8 vs. 3.7 appointments, respectively). As age may differ between groups (i.e., older patients may have used hearing aids for longer durations), a one-way analysis of variance assessing the impact of past experience on age was completed. However, there was no significant differences of age between users based on previous experience, *F*(1, 47) = 0.95, *p* = .57.

### Device Factors

The majority of patients were fit with RIC devices (58.7%) and devices with rechargeable batteries (83.8%; see Table 2). Most patients were fit with standard (38.1%) or advanced (32.3%) technology, while a smaller proportion were fit with basic- (12.1%) or premium- (17.5%) level devices.

Analyses did not identify an effect of style of technology or battery type on number of follow-up appointments (see Table 2). An effect of level of technology was identified for those using standard technology (β = .294, *p* = *.*017). Those with standard technology attended one more appointment (4.5 appointments) relative to those patients who were fit with basic technology (3.5 appointments), *t*(231) = 2.29, *p* = .022, and premium technology (3.6 appointments), *t*(231) = 2.22, *p* = .026. No other significant differences between technology levels were noted (*p* > .05). Dispensing an accessory impacted appointment attendance (β = .216, *p* = .019*).* Patients without an accessory attended fewer appointments relative to those with an accessory, *t*(231) = −2.25, *p* = .025; 3.9 versus 4.8 appointments, respectively. To evaluate whether accessory dispensing was associated with hearing loss severity, worse-ear PTA by accessory status was examined. Worse-ear PTA differed significantly between those who received accessories versus those who did not (72.12 ± 25.12 vs. 58.23 ± 19.40 dB HL; *t* = −3.88, *p* = .0001).

### Payment Factors

In our patients, only 37 (16.6%) did not have any insurance coverage and 84 (37.7%) had full insurance coverage (see Table 3). Analyses found a significant effect of insurance coverage and OOP costs on number of appointments attended (see Table 3). Postestimation testing identified a significant difference between no coverage and full coverage (*t* = 2.39, *p* = .017), while no other significant differences were noted between groups (*p* > .05). On average, patients without coverage attended 4.6 appointments while those with full coverage attended 3.6 appointments.

There was significant overlap of total OOP costs between patients with partial coverage (range: $199–$4,998) and those without any insurance benefits (range: $1,380–$5,936; see Table 3). Thus, additional analyses were completed assessing impact of increased OOP costs on follow-up appointments not accounting for insurance coverage. Overall, we identified an effect for those with OOP cost totals from $1,001 to $2,000 (β = .243, *p* = .018) and those who spent $2,000 or more (β = .271, *p* = .002). There was a trend that increasing OOP costs led to higher service utilization as patients with OOP costs from $1,001–$2,000 and over $2,000 attended 4.5 and 4.8 appointments, respectively, while those without any OOP costs and costs under $1,000 attended 3.5 appointments. Postestimation testing indicated that those with no OOP costs or costs under $1,000 were not significantly different from each other, *t*(231) = −0.60, *p* = .551, nor were those with $1,000–$2,000 and over $2,000, *t*(231) = 0.61, *p* = .542. Differences between groups were noted on postestimation comparisons between those groups with costs under $1,000 and those with costs over $1,000 (*p* < .018).

Level of technology and OOP cost display known interdependencies as higher levels carry a higher cost, and insurance coverage may influence the level of technology purchased (i.e., a patient may select the highest level or highest cost hearing aid that insurance will cover). As the distributions between OOP costs and level of technology were unbalanced, differences in distribution of insurance coverage and OOP costs by technology level dispensed were assessed using chi-squared tests. The distribution of technology level varied based on insurance coverage (χ^2^ = 135.65, *p* < .001) and OOP cost (χ^2^ = 99.65, *p* < .001). The majority of patients (89%) with full coverage (i.e., no OOP cost) selected an advanced- or premium-level technology with only 11% receiving standard level technology. Most patients with partial coverage were dispensed basic (22%) or standard (61%) technology with a minority (16%) receiving advanced- or premium-level technology. Those without coverage were largely dispensed basic (43%) or advanced technology (32%), while a small minority had premium-level technology (19%).

Similarly, those who paid $1–$1,000 were all dispensed basic or standard technology. The majority of patients (80%) with an OOP cost between $1,000 and $2,000 had standard technology. A wider spread was seen for patients with an OOP cost $2,000 or higher, with a relatively even distribution between standard (35%), advanced (37%), and premium (22%) technology.

## Discussion

On average, patients attended four follow-up appointments after hearing aid fitting within the first year with the majority attending three to five appointments. A significant influence of patient-specific demographic and audiologic factors on number of appointments was not identified. Thus, clinicians and adult patients can expect approximately three to five follow-up visits during the first year after fitting, regardless of age, degree of hearing loss, or language background. However, patients with a higher OOP cost and those using standard-level technology attended on average one more follow-up appointment. Patients fit with standard technology tended to be those with a higher OOP cost and partial insurance coverage. This may reflect an impact of cost on satisfaction and subsequent seeking of professional services.

While a statistically significant impact of age on follow-up appointment utilization was identified, this was a small effect, suggesting that, in general, per every decade increase in age, .06 more follow-up appointments occurred. Additionally, an effect of past experience was identified in that those with 10 or more years of prior hearing aid use attended more appointments than new users. While duration of prior experience may be related to age, significant differences were not detected between our groups, suggesting that this was not an influence. This may suggest that long-time users have expectations borne from the previous use of hearing aids that the new user would not. They might want to have the new devices sound similar to the previous devices, requiring additional visits for fine tuning in comparisons to new users without a prior perceptual benchmark.

Poorer WRS or greater degrees of hearing loss may be expected to lead to additional follow-up appointments due to anticipated increased difficulty with speech recognition with their hearing aids (Chang et al., 2012; Wang et al., 2021, 2022). However, audiometric factors, including PTA or WRS, did not impact utilization of clinic services.

In general, most device-specific features did not impact the number of follow-up appointments attended by patients. An effect of a co-occurring accessory being dispensed (e.g., remote microphone) was identified, as those with at least one accessory had a higher number of appointments. Accessories are often dispensed to patients with greater hearing loss in order to increase the signal-to-noise ratio (Thibodeau, 2014), and accessory recipients in our sample demonstrated poorer hearing (higher worse-ear PTA). Nevertheless, worse-ear PTA was not an independent predictor of appointment utilization in adjusted models, indicating that the observed association may reflect broader clinical complexity and counseling demands rather than audiometric severity alone. Use of these devices can take substantial time for counseling and instruction (Hermawati & Pieri, 2020). Increased need for clinician time is therefore expected, and patients should be counseled appropriately.

In our sample, device and payment factors displayed significant interdependencies. Those patients with standard-level technology on average attended approximately one more appointment relative to patients who were fit with premium-level technology. Past evidence suggests that premium-level technology may provide modest improvements in satisfaction and speech understanding (Hausladen et al., 2022; Johnson et al., 2016). Thus, premium-level users may have been less inclined to return to clinic for follow-up appointments due to a greater perceived benefit or belief that performance was optimized with premium technology. Those patients using basic technology also had fewer follow-up appointments; thus, when considering cost relative to benefit, this may suggest that these users may have been less likely to return to the clinic as they perceived that their benefit was maximized relative to the level of technology received.

While level of technology and post-fitting follow-up care needed may reflect perceived benefit, distribution of technology level varied on insurance coverage and OOP cost. This suggests that other patient-specific factors outside of level of technology influenced follow-up care. For example, those with basic technology may have attended fewer appointments due to barriers to seeking care such as required time off work, transportation, and so forth (Iwahashi et al., 2015; Solheim et al., 2012, 2018). Although socioeconomic barriers may impact ability to attend follow-up appointments, these were not directly measured during this retrospective study.

Additionally, the vast majority of patients with standard technology level who attended a higher number of appointments, had partial insurance coverage (62 out of 85) and an OOP cost from $1,000–$2,000. As analyses also revealed that those with partial or no coverage and OOP costs over $1,000 dollars attended more follow-up appointments, the finding that standard technology level users attended more appointments likely reflects their OOP cost. Past evidence suggests that higher cost is associated with greater perceived benefit, but not higher satisfaction (Bannon et al., 2023; Kim et al., 2022). As such, patients with a higher cost may have perceived less benefit relative to the cost of the devices and, thus, been more likely to return more often to maximize their benefit.

Although this study was conducted in a university audiology clinic, the patterns observed are likely relevant to other clinics that dispense hearing aids and seek to improve patient satisfaction and improve clinic efficiency. Counseling on the purpose and value of follow-up appointments can improve adherence to attending follow-up appointments (Khan et al., 2023; Solheim et al., 2012), and the present findings can be used to provide patients with evidence-based recommendations on anticipated number of appointments. Adequate follow-up can improve short-term satisfaction (Solheim et al., 2018), yet patients experience ongoing frustrations after hearing aid acquisition and are reluctant to seek help from their clinician (Bennett et al., 2019). Providing clear informational counseling about anticipated follow-up and troubleshooting appointments may help normalize help-seeking and encourage patients to return when problems arise.

Additionally, historically, amplification has been provided in a bundled service model in which devices and professional services are combined into a single cost. An increasing number of audiology clinics have adopted an unbundled model in order to facilitate cost transparency and promote adoption of amplification (Fifer, 2020; Picou et al., 2024), yet many clinics have not fully moved to this approach. Unbundled pricing structures may introduce inequities if specific patient groups systematically over- or underutilize services; however, the current findings suggest no clear inequalities in the number of appointments based on patient demographics. These data may help clinics estimate typical follow-up patterns when structuring service packages or setting fees for unbundled follow-up care to better align clinic resources with expected patient needs

From an operational perspective, these findings may help clinics translate expected follow-up utilization into staffing, scheduling, and service-delivery decisions. Knowing that most patients require several post-fitting visits and that follow-up needs are modestly higher for certain subgroups, practices may benefit from proactive triage—such as scheduling an earlier first follow-up and reserving additional time for counseling and device management when accessories are dispensed. Many components of post-fitting support (e.g., device orientation, accessory setup and connectivity checks, and routine troubleshooting) may be well suited to trained support personnel (e.g., audiology assistants/technicians), allowing audiologists to focus on more complex clinical decision making.

### Limitations and Future Directions

The clinic included represents a unique university-affiliated audiology setting in southern Arizona. Approximately 30% of the patients are Spanish speaking, and the clinic has bilingual staff. The need to use an interpreter may influence results. All services included involvement of graduate students who typically require more time to provide the same service than an experienced professional. Additionally, potential variation in follow-up scheduling practices across student clinicians and supervising audiologists could not be examined. As student clinicians rotated across academic terms and supervising clinician varied based on clinician availability, it was not feasible to attribute follow-up patterns to a specific clinician or dyad. Consequently, some variability in the number of visits may reflect unmeasured differences in clinician practice style.

The finding of unique interactions between OOP cost, technology level, and insurance coverage is likely not unique to the included population and should be further explored in additional populations. Particularly, inclusion of a measure of outcome measures, including benefit and/or satisfaction, would allow insight into how OOP cost influences device satisfaction and seeking additional services in the clinic. Inclusion in future studies would allow direct comparisons between outcomes and clinic utilization.

Patient outcomes or satisfaction were not directly measured through the use of standardized questionnaires, thus hindering insight into potential relationships between outcomes and follow-up appointments needed. Future efforts should track both subjective outcomes and appointment usage in the same patients to assess potential interactions.

Additionally, this retrospective study only assessed follow-up care over a 1-year period. Patient- or device-specific differences may become more apparent over a longer time period. As this was a retrospective study, assessing the number of appointments attended was the main focus. Future prospective studies should assess both the number and total duration of appointments to better determine professional service usage and also document reason for follow-up and/or specific patient concerns.

### Conclusions

This retrospective chart review characterized real-world follow-up patterns in adult hearing aid users in a university-affiliated audiology clinic. Most patients attended approximately three to five follow-up appointments in the year after fitting, and demographic and audiometric variables had minimal impact on service utilization. Device and payment factors, particularly the level of technology and OOP cost, showed modest associations with higher follow-up use. These findings suggest that clinicians can provide patients with simple, evidence-based expectations about typical follow-up needs and can account for slightly higher service use among patients with higher OOP costs in their clinical decision making. More broadly, the data can inform clinic decisions about follow-up protocols, scheduling, and structuring bundled or unbundled service packages for adult hearing aid care.

## Data Availability Statement

The data sets generated and/or analyzed during the current study are available from the corresponding author on reasonable request.
